# The effects of student bullying on non-suicidal self-injurious behavior in rural adolescents: the chain-mediated effects of alexithymia and ruminate thinking

**DOI:** 10.3389/fpsyg.2024.1483408

**Published:** 2024-12-16

**Authors:** Jing Wen, Qinghong Xu, Yongzhi Jiang, Min Li

**Affiliations:** ^1^School of Education, Inner Mongolia Minzu University, Tongliao, China; ^2^Inner Mongolia Student Bullying Prevention Research Center, Tongliao, China; ^3^Inner Mongolia Ethnic Education and Psychological Development Research Base, Tongliao, China; ^4^School of Foreign Languages, Yulin University, Yulin, China

**Keywords:** student bullying, non-suicidal self-injury, alexithymia, rumination, goal progression theory, gender differences

## Abstract

Bullying among students is a significant risk factor for non-suicidal self-injury (NSSI), which can adversely impact the physical and mental health development of individuals. To explore the mechanisms by which student bullying affects adolescent non-suicidal self-injury, 701 adolescents were selected as participants. The assessment tools included the Bullying Questionnaire, Toronto Alexithymia Scale, Ruminative Responses Scale, and Adolescents Non-suicidal Self-injury Assessment Questionnaire, which were used to measure the experiences of bullying, levels of alexithymia, rumination tendencies, and the severity of non-suicidal self-injury, respectively. This study aimed to examine whether there is a chain mediation effect of alexithymia and rumination in the relationship between student bullying and non-suicidal self-injury. The results indicated that: (1) Student bullying has a significant positive impact on adolescent non-suicidal self-injury (63.62%) and indirectly influences it through alexithymia and rumination (12.69%); (2) There are gender differences in the chain mediation effect between student bullying and non-suicidal self-injury, with the chain mediation effect of rumination and alexithymia being significantly greater in females (0.12) compared to males (0.06). This study not only provides a cognitive-emotional perspective and a gender difference perspective on the effects of bullying on adolescent non-suicidal self-injury but also focuses on rural adolescents, who often face more physical and mental health issues. Therefore, the conclusions enrich the understanding of the complex mechanisms between student bullying and non-suicidal self-injury, offering new theoretical guidance for preventing bullying and intervening with victims of bullying.

## 1 Introduction

Non-suicidal self-injury (NSSI) refers to the intentional harm inflicted on one’s own body without the intent to die (Ahn et al., 2021). Adolescents are a high-risk group for NSSI, with the behavior peaking between the ages of 15 and 17, and there is a noticeable upward trend in NSSI rates (Plener et al., 2015). Previous studies indicate that the one-year prevalence of NSSI among Chinese adolescents is 15.5% (Tang et al., 2011). NSSI is an important risk factor for predicting suicidal behavior (Tang et al., 2011; Wilkinson et al., 2011; Asarnow et al., 2011), meaning that individuals with a broader history of self-injurious behavior are more likely to attempt suicide. Joiner posits that once adolescents view self-injury as a coping mechanism for stress, they may be more inclined to engage in such behavior more frequently. This attribution can reinforce self-injurious behaviors and exacerbate the relationship between NSSI and suicidal behavior (Joiner, 2005). The escalating suicide rates severely impact the maintenance of social harmony and stable development; thus, understanding the mechanisms underlying NSSI is crucial for reducing suicide rates.

In recent years, there has been a significant increase in research examining the relationship between NSSI among adolescents and bullying experiences, clearly identifying that experiences of bullying are an important risk factor triggering non-suicidal self-injurious behavior (Ayano et al., 2021). Bullying is characterized by an attack from a stronger party on a weaker one and involves repetition, power imbalances, and intentional harm. It primarily includes four forms: physical bullying, relational bullying, verbal bullying, and cyberbullying. Survey data indicate that bullying is a widespread phenomenon globally, with approximately one-third of children having experienced bullying. In China, the incidence of bullying victimization in primary and secondary schools reaches as high as 25.8% (Olweus, 2013; Tan, 2023). The impacts of bullying on individual mental health are profound; it not only causes immediate psychological trauma to victims but can also lead to serious negative effects on their long-term development. Studies have shown that the rate of NSSI among bullying victims is higher than that among non-victims (Li et al., 2020; Lee et al., 2021). Furthermore, numerous scholars have confirmed a significant positive correlation between bullying and NSSI, indicating that adolescents who experience higher levels of bullying are more likely to exhibit self-injurious tendencies (Yang et al., 2023; Wen et al., 2023; Van Geel et al., 2015; Huang et al., 2022). General strain theory suggests that when one is unable to resolve or adapt to the negative emotions caused by violent events, such feelings may lead an individual to take a series of deviant behaviors in order to relieve or eliminate such feelings (Agnew, 1992). Based on these findings, this study proposes Hypothesis H1: Adolescent experiences of bullying significantly positively predict their engagement in non-suicidal self-injury.

Existing research has indicated that one of the significant predictive variables for non-suicidal self-injury (NSSI) among adolescents is alexithymia, with its importance increasingly recognized (Ruan et al., 2024). Alexithymia refers to an individual’s insufficient ability to recognize, process, and regulate emotions, specifically manifested as difficulty in identifying and describing feelings, distinguishing emotions from bodily sensations, and a lack of fantasy and extraverted thinking traits (Taylor, 2000). Studies have shown that alexithymia has a direct impact on adolescents’ non-suicidal self-injurious behavior (Zhang et al., 2023). For example, one study found that individuals with high levels of alexithymia are more likely to engage in NSSI, suggesting that such behavior may serve as an externalizing coping mechanism (Paivio and McCulloch, 2004).

Moreover, it is noteworthy that alexithymia is highly correlated with experiences of bullying. Specifically, Levantini et al. found that victims of bullying scored higher on measures of alexithymia compared to non-victims (Levantini et al., 2023). Overall, several studies have consistently identified a relationship between alexithymia and various severe outcomes, including trauma-related stress in adolescents and preadolescents, deliberate self-injury, as well as internalizing and externalizing problems (Guzzo et al., 2014; Garisch and Wilson, 2010; Prino et al., 2019). These findings provide compelling arguments for the role of alexithymia as a mediator between bullying and non-suicidal self-injury.

These arguments are rooted in a fundamental theoretical framework, particularly from the perspective of social mechanisms of alexithymia. This theory posits that certain adverse social factors encountered during child development can diminish socialization levels and impair emotional regulation abilities, leading to maladaptive behaviors (Honkalampi et al., 2004). This implies that Honkalampi and colleagues emphasize the role of external social factors in the development of alexithymia, where bullying, as a typical social trauma event, can significantly affect an individual’s ability to express and identify emotions. In the context of bullying, victims often face difficulties in emotional suppression and expression, which results in ineffective regulation of their negative emotions and internal evaluations. This emotional dysregulation further exacerbates the psychological distress experienced by the victims and may prompt them to engage in NSSI as a coping strategy.

Based on the aforementioned theoretical perspectives and analyses, this study proposes that to fully understand the impact of bullying on non-suicidal self-injury, it is essential to explore the direct and indirect relationships between bullying experiences, alexithymia, and NSSI, thereby establishing a path relationship model among the three factors. Accordingly, this research posits the following hypothesis H2: Alexithymia mediates the relationship between bullying victimization and non-suicidal self-injury among adolescents. This hypothesis not only unveils the underlying connections between bullying and NSSI but also emphasizes the crucial role of alexithymia in this process.

Furthermore, we note that rumination is an important mediating variable influencing the relationship between bullying and non-suicidal self-injury (NSSI) among students (Wang, 2021). Rumination refers to the tendency of individuals to continually focus on negative experiences following adverse life events, persistently thinking about the causes and consequences of these events, along with the accompanying negative emotions, while failing to actively seek solutions. It represents an excessive preoccupation with problems rather than a proactive coping approach (Nolen-Hoeksema, 1991). Individuals who experience bullying often struggle with unresolved stress, making them more likely to become immersed in negative emotions, negative thoughts, and memories related to the adverse events, thereby intensifying their rumination (Chu et al., 2019).

Additionally, studies have shown a significant positive correlation between bullying and rumination, indicating that individuals who experience bullying more frequently tend to have stronger ruminative responses (Chu et al., 2019; Wang et al., 2023). According to the stress response model, if the outcome of a stressful event is negative, individuals are likely to ruminate over that negative outcome (Robinson and Alloy, 2003). Therefore, when victims of bullying struggle to resolve or adapt to the situation, they may continuously reflect on the negative consequences of being bullied.

Moreover, investigations have revealed a close relationship between rumination and non-suicidal self-injury (Voon et al., 2014). Persistent rumination can exacerbate an individual’s negative experiences, heighten their focus on distress, and ultimately lead to NSSI as a means of temporarily alleviating that pain. Consequently, individuals with high levels of rumination are more likely to engage in non-suicidal self-injurious behavior (Fu et al., 2024). Previous research has also identified rumination as a significant predictor of NSSI (Bjärehed and Lundh, 2008). Selby et al. further argued that NSSI serves as a painful yet non-lethal method for diverting attention away from ruminative thoughts (Selby and Joiner, 2010).

In summary, these findings highlight the critical role of rumination as a mediating factor between bullying experiences and non-suicidal self-injury, suggesting that individuals who are bullied may resort to self-injury as a maladaptive coping mechanism fueled by their ruminative tendencies.

Given the significant role of rumination in the relationship between experiences of bullying and non-suicidal self-injury (NSSI), we can understand bullying as a negative stressor that, when individuals are unable to cope with it effectively, leads to persistent contemplation of its negative consequences. This can create a vicious cycle of rumination and negative emotions, further exacerbating negative thoughts and deepening emotional distress. Individuals who are bullied often exhibit elevated levels of rumination (Monti et al., 2017), and mild stimuli are insufficient to divert their attention from negative outcomes. As a result, they may resort to more intense strategies, such as engaging in NSSI, to shift their focus towards bodily pain and the stimulation of blood, using it as a method to distract themselves. This approach can rapidly alleviate the negative effects of bullying on mental health and may even lead to NSSI becoming a habitual coping mechanism for individuals (Selby and Joiner, 2010). Given the adverse impacts of bullying on mental health, this study posits that bullying is not only directly associated with NSSI but may also indirectly promote NSSI behaviors through the mediation of rumination. Hence, based on this analysis, we propose Hypothesis H3: Rumination mediates the relationship between bullying and non-suicidal self-injury among adolescents.

In thorough investigations of previous research, both rumination and alexithymia have been identified as influential predictors of non-suicidal self-injury (Borrill et al., 2009). This finding provides support for constructing a chain mediation model between bullying and NSSI. Individuals with alexithymia typically exhibit difficulties in recognizing and expressing emotions, as well as social withdrawal, which limits their information-processing capabilities. This limitation is particularly pronounced among adolescents who have experienced bullying, as they often struggle to effectively cope with or adapt to the various consequences of bullying events. This not only exacerbates their psychological burden but also leads to further difficulties in information recognition. From the perspective of goal progression theory, rumination is viewed as a form of self-regulated thinking that arises when individuals perceive significant goals being obstructed, resulting in persistent thoughts about the situation (Martin et al., 2003). Such individuals tend to continuously focus on negative situations and outcomes (Di Schiena et al., 2011).

Some scholars have found a significant positive correlation between alexithymia and rumination, implying that the severity of alexithymia is associated with higher levels of rumination (Liu et al., 2022). Additionally, in a study specifically targeting patients with depression, it was confirmed that depressed individuals with alexithymia exhibited higher levels of rumination compared to those without alexithymia (Du and Dong, 2019). This finding further validates the close relationship between alexithymia and rumination. Furthermore, some research has indicated that alexithymia and anger rumination play a chain-mediation role in the relationship between social trauma and suicidal ideation (Wang and Zhang, 2023).

Based on these significant findings, while consistent outcomes have been observed in the direct relationship between bullying and NSSI, it raises the question of whether this consistency extends to the chain mediation of alexithymia and rumination. Therefore, this study proposes Hypothesis H4: Alexithymia and rumination serve as chain mediators in the impact of bullying on NSSI among adolescents. The introduction of this hypothesis not only helps to deepen our understanding of the complex mechanisms linking bullying and adolescent NSSI but also provides important theoretical support for developing effective prevention and intervention measures.

During adolescence, students develop a greater sense of independence, and their emotions often become unstable. Social gender theory posits that females, characterized by their sensitivity, emotionality, and nurturing traits, often display deeper empathy and a propensity to help others. In contrast, males typically exhibit boldness, competitiveness, aggression, and rationality. Individuals internalize and conform to socially prescribed gender role expectations, which subsequently govern their behaviors (Espelage and Swearer, 2004). When confronted with bullying, females are more likely to demonstrate emotional internalization and have lower self-esteem, exhibiting higher levels of negative thinking when compared to males (Butler and Nolen-Hoeksema, 1994). Males, due to their competitive nature, are more likely to get involved in bullying scenarios, and they tend to adopt proactive aggressive coping strategies when facing bullying (Xia et al., 2023). Furthermore, the positive traits attributed to males, such as strength and independence, can somewhat mitigate the harm caused by bullying. Therefore, there may be significant gender differences in the chain mediation effect observed between bullying and non-suicidal self-injury (NSSI). This leads to the formulation of Hypothesis H5: There exists a gender difference in the chain mediation model of the impact of bullying on non-suicidal self-injury.

In summary, this research focuses on adolescents as the study population, utilizing emotional cascade theory, the social mechanisms of alexithymia, and goal progression theory to substantiate and explore the empirical findings. It aims to investigate the mediating roles of alexithymia and rumination in the relationship between bullying experiences and non-suicidal self-injury among rural adolescents. The study seeks to further examine the explanatory capacities of these theories and prior findings regarding the mechanisms involved in the relationships among bullying, non-suicidal self-injury, and rural adolescents, thereby providing valuable insights for reducing the incidence of NSSI in this demographic.

Integrating the hypotheses H1, H2, and H3, we have constructed a chain mediation model, as illustrated in Model 1. Although a considerable amount of research has been conducted on adolescent non-suicidal self-injury, most studies focus on urban youth. Rural adolescents, affected by more complex environments, are likely to face a greater array of psychological issues. This study not only provides a unique cognitive-emotional perspective and an in-depth exploration of gender differences regarding bullying’s impact on adolescent NSSI but also specifically targets the rural adolescent group, which faces more pronounced physical and mental health challenges, conducting a specialized investigation into their experiences.

By exploring the complex mechanisms surrounding bullying and non-suicidal self-injury, we gain a deeper understanding of the causes and mechanisms underlying these behaviors, as well as the emotional and cognitive processes at play for students. Moreover, adolescents experiencing bullying are at risk for individual and societal developmental issues; thus, this research will aid in identifying high-risk individuals and inform future comprehensive interventions and therapeutic strategies from the emotional-cognitive and gender difference perspectives. This approach aims to alleviate the suffering of bullying victims and prevent the occurrence of non-suicidal self-injury behaviors, which is vital for safeguarding adolescent mental health, maintaining campus harmony, and promoting healthy societal development.

## 2 Research methodology

### 2.1 Participants

Adolescent students from six high schools in Tongliao City, Inner Mongolia, participated in a questionnaire survey. A total of 826 questionnaires were distributed, and after careful screening, 125 were excluded for clearly insufficient responses, resulting in 701 valid participants, yielding an effective response rate of 84.7%. Details of the valid participants are presented in Table 1. Participants were from several schools in Naihanqi of Tongliao City, with 550 participants (78.5%) from rural areas and 133 participants (19.0%) from urban areas. Among the participants, 187 (26.7%) held class leadership positions, while 493 (70.3%) did not. Regarding the only-child designation, 225 participants (32.1%) were only children, while 464 (66.2%) were not. In terms of family structure, 563 participants (80.3%) came from intact families, 67 (9.6%) from single-parent families, and 56 (8.0%) from blended families. The gender distribution was roughly balanced, with a slight majority of females (51.16%), while males comprised 338 individuals (48.84%). As for grade levels, there were 63 participants (9.0%) in the first year of junior high school, with an average age of about 13 years; 118 participants (16.8%) in the second year, with an average age of 14 years; 144 participants (20.5%) in the third year, with an average age of 15 years; 150 participants (21.4%) in the first year of senior high school, with an average age of approximately 16 years; 143 participants (20.4%) in the second year, with an average age of around 17 years; and 74 participants (10.6%) in the third year, with an average age of about 18 years.

**Figure 1:**
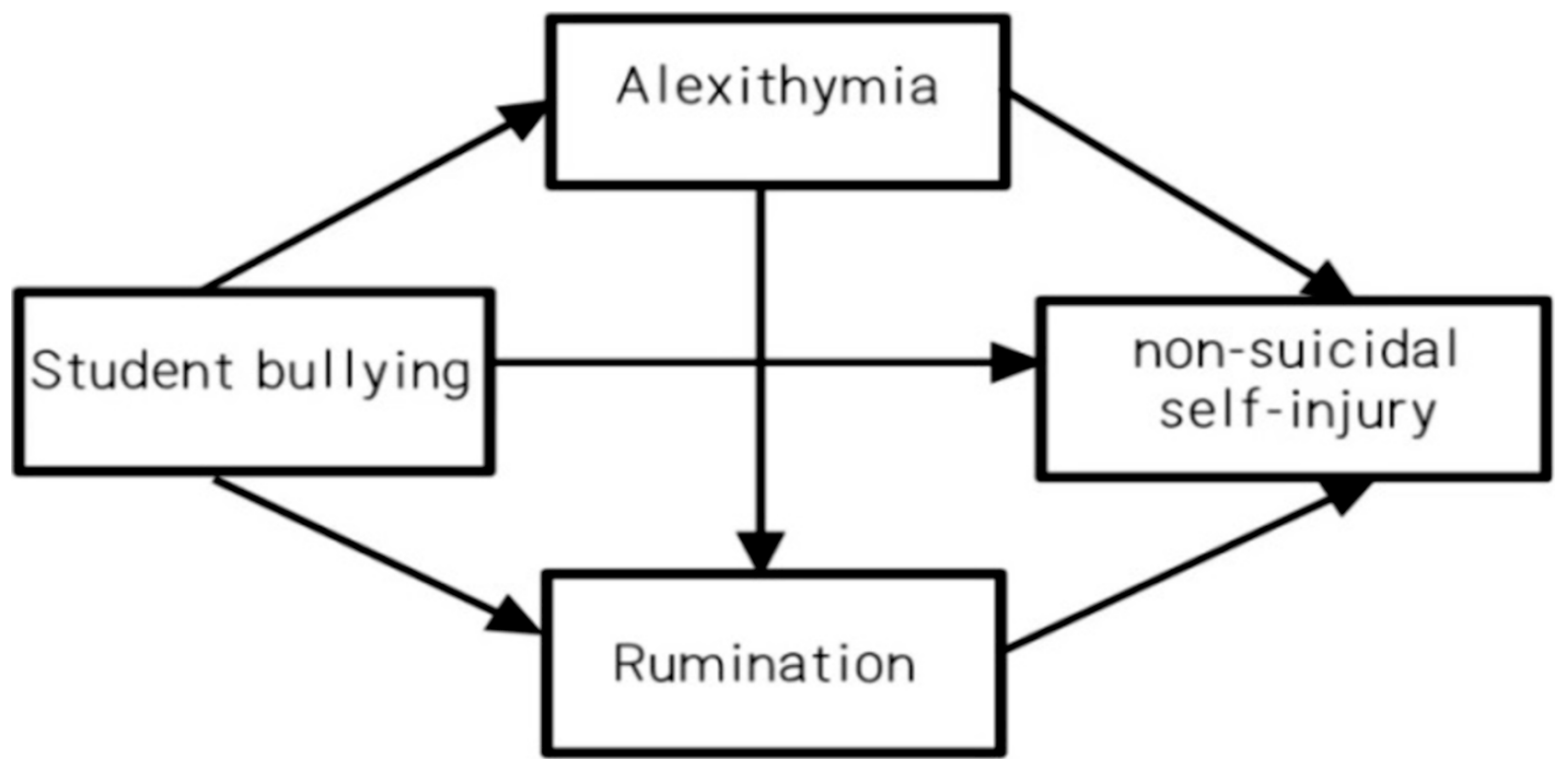
The chain mediation model assumes a path graph.

**Table 1:** Statistics on the number of boys and girls in each grade.

Grade	Male	Female	Total
First grade	27	36	63
Second grade	69	49	118
Third grade	72	72	144
Freshman year	80	70	150
Sophomore year 6	66	77	143
Senior year	24	50	74
Total	338	354	692

Data collection and entry for this study occurred from November 2023 to January 2024.

### 2.2 Inclusion and exclusion criteria

#### 2.2.1 Inclusion criteria

1. Ages between 12 and 18 years;
2. Ability to properly understand the contents of the questionnaire and willingness to cooperate with the survey;
3. Consent from the participants and their guardians.

#### 2.2.2 Exclusion criteria

1. Presence of neurological or other mental disorders;
2. Substance or alcohol dependency;
3. Conditions such as epilepsy, brain injury, or intellectual disabilities;
4. Recent exposure to significant stressful events;
5. Hearing, speech, or other communication impairments that affect normal interaction;
6. Inability to cooperate with the surveyors.

### 2.3 Research tools

Item Packing Note: The following four scales were modeled with latent variables in the analysis of the fitted model in question items packaged three or four adjacent to each other when using AMOS.

#### 2.3.1 Bullying questionnaire

This study adopted the bullying scale from the Olweus Bullying Questionnaire revised by Zhang Wenxin and other scholars (1999) (Zhang and Wu, 1999). The questionnaire asked the subjects about the frequency of school bullying from their classmates in the past 3 months, and the questionnaire consisted of 6 questions. It was divided into three dimensions: verbal bullying (2 questions about some classmates calling me unpleasant nicknames or making fun of and being sarcastic to me), physical bullying (2 questions about some classmates hitting, kicking, pushing, bumping into me, or threatening me), and relational bullying (2 questions about some classmates spreading some rumors about me through cell phones or computers, and trying to make other people dislike me). Likert’s 5-point scale was used, with one meaning “it did not happen,” 2 meaning “it only happened once or twice,” 3 meaning “two or three times a month,” 4 meaning “about once a week,” and 5 for “several times a week.” The higher the total score, the higher the individual’s level of bullying. In this study, the bullying questionnaire factor fit model was good: *χ* /df = 3.18, RMSEA = 0.06, SRMR = 0.02, CFI = 0.99, TLI = 0.97, and Cronbach’s alpha coefficient was 0.81.

#### 2.3.2 Adolescents’ non-suicidal self-injury assessment questionnaire

Developed by Yuhui Wan et al. to obtain information on the occurrence of 12 self-injurious behaviors in the study population in the last year. The total questionnaire was divided into functional and behavioral questionnaires (Wan et al., 2018). The behavioral questionnaire consisted of 12 entries and was divided into two dimensions using Likert’s 5-point scale: the group with no apparent tissue damage and the group with apparent tissue damage. The no obvious tissue damage included seven questions, such as intentionally scratching hair, pinching, and biting oneself. The no apparent tissue damage included five questions, such as intentionally cutting oneself and intentionally rubbing the skin with something to make it bleed or bruise. ^2^In this study, the non-suicidal self-injury questionnaire factor fit model was good: *χ*/df = 5.99, RMSEA = 0.08, SRMR = 0.05, CFI = 0.92, TLI = 0.90, and Cronbach’s *α* was 0.87.

#### 2.3.3 Toronto alexithymia scale

The Toronto Alexithymia Scale (TAS) was developed by Bagby et al. and revised by Jin-Yao Ant et al. (Yi et al., 2003). The scale consists of 20 questions divided into three dimensions: difficulty in identifying emotions (e.g., I am often confused about what kind of feelings I have. 7 questions), difficulty in expressing emotions (e.g., I can easily describe my feelings. 5 questions), and extroverted thinking (e.g., I prefer to talk to others about their daily activities rather than their feelings. 8 questions). A 5-point Likert scale was used, with higher scores indicating higher levels of dysfunction in describing feelings. In this study, the Toronto Alexithymia Scale factor fit model was good: *χ*/df = 5.50, RMSEA = 0.08, SRMR = 0.08, CFI = 0.85, TLI = 0.83, and Cronbach’s alpha coefficient was 0.75.

#### 2.3.4 Ruminative responses scale (RRS)

Prepared by Nolen-Hoeksema, revised into Chinese by Han and Yang (2009), it consists of 22 questions divided into three dimensions: symptomatic rumination (e.g., I often think about how lonely I am. 12 questions), obsessive-compulsive thinking (e.g., I often think about what I did to cause this. 5 questions), and introspective thinking (e.g., I often think alone about why this is the case. 5 questions). A 4-point Likert scale was used, with higher scores indicating a greater tendency to ruminate. The ruminative thinking questionnaire factor fit model was good: χ/df = 3.96, RMSEA = 0.07, SRMR = 0.04, CFI = 0.91, TLI = 0.90, and the internal consistency alpha coefficient of this scale in this study was 0.93.

### 2.4 Procedures

This study was approved by the Research Ethics Committee of Inner Mongolia University for Nationalities. After establishing contact with teachers from two middle schools in Naiman Banner, Tongliao City, the researcher introduced the purpose and content of the study. After acquiring their consent, the teachers in the local school used the paper version of the questionnaire to conduct the whole class test in their spare time, and the students completed the questionnaire anonymously. Written informed consent and assent forms were acquired from the participants and their legal guardians/relatives. Participation was voluntary, and confidentiality was guaranteed. The data collectors consisted of trained researchers who ensured the standardization of the data collection process. Completing the self-report questionnaire took approximately 30 min.

### 2.5 Data analysis

Descriptive analysis of the data was conducted using SPSS 25.0 software. The correlations between variables were assessed using Pearson product–moment correlation analysis. Structural equation modeling (SEM) was constructed using AMOS 28.0 software, and the mediation effects along with gender difference tests were examined using the bias-corrected nonparametric percentile Bootstrap method. A 95% confidence interval that does not include zero indicates a significant mediation effect. Statistical significance was defined at *p* < 0.05.

## 3 Results

### 3.1 Common method bias test

Using only the questionnaire method in this study may create the problem of common method bias, which was controlled by emphasizing confidentiality and positive and negative scoring during the test administration. A common method bias test was also conducted. Validated factor analysis was used, and the results showed that the one-way model fit was poor: *χ*/df = 22.46, RMSEA = 0.18, SRMR = 0.13, CFI = 0.64, and TLI = 0.57, which suggests that common method bias is not a severe problem and that the effect of common method bias can be ruled out.

### 3.2 Descriptive statistics and correlation analysis

Table 2 presents the means, standard deviations, and correlation coefficients for the various variables. The analysis revealed significant positive correlations among bullying experiences, alexithymia, rumination, and non-suicidal self-injury (NSSI) in pairs. This indicates that the more frequently students experience bullying, the more difficult it is for adolescents to recognize, express, and regulate their emotions. Additionally, the severity of bullying correlates with the severity of adolescents’ NSSI behaviors; those who find it more challenging to identify and express emotions, as well as exhibit more severe alexithymia, demonstrate higher levels of rumination. Furthermore, students who engage in rumination more frequently tend to have a higher occurrence of NSSI. The relationships among these variables support the further testing of the proposed hypotheses Figure 1.

**Table 2:** Descriptive statistics and correlation of main variables.

Variable	M ± SD	Student bullying	Alexithymia	Rumination	Non-suicidal self-injury
Student bullying	1.14 ± 0.31	1.00			
Alexithymia	55.55 ± 9.58	0.13**	1.00		
Rumination	42.45 ± 12.01	0.20***	0.55***	1.00	
Non-suicidal self-injury	13.56 ± 3.78	0.22***	0.31***	0.39***	1.00

*p < 0.05, **p < 0.01, ***p < 0.001.

### 3.3 Tests for mediating effects

**Figure 2:**
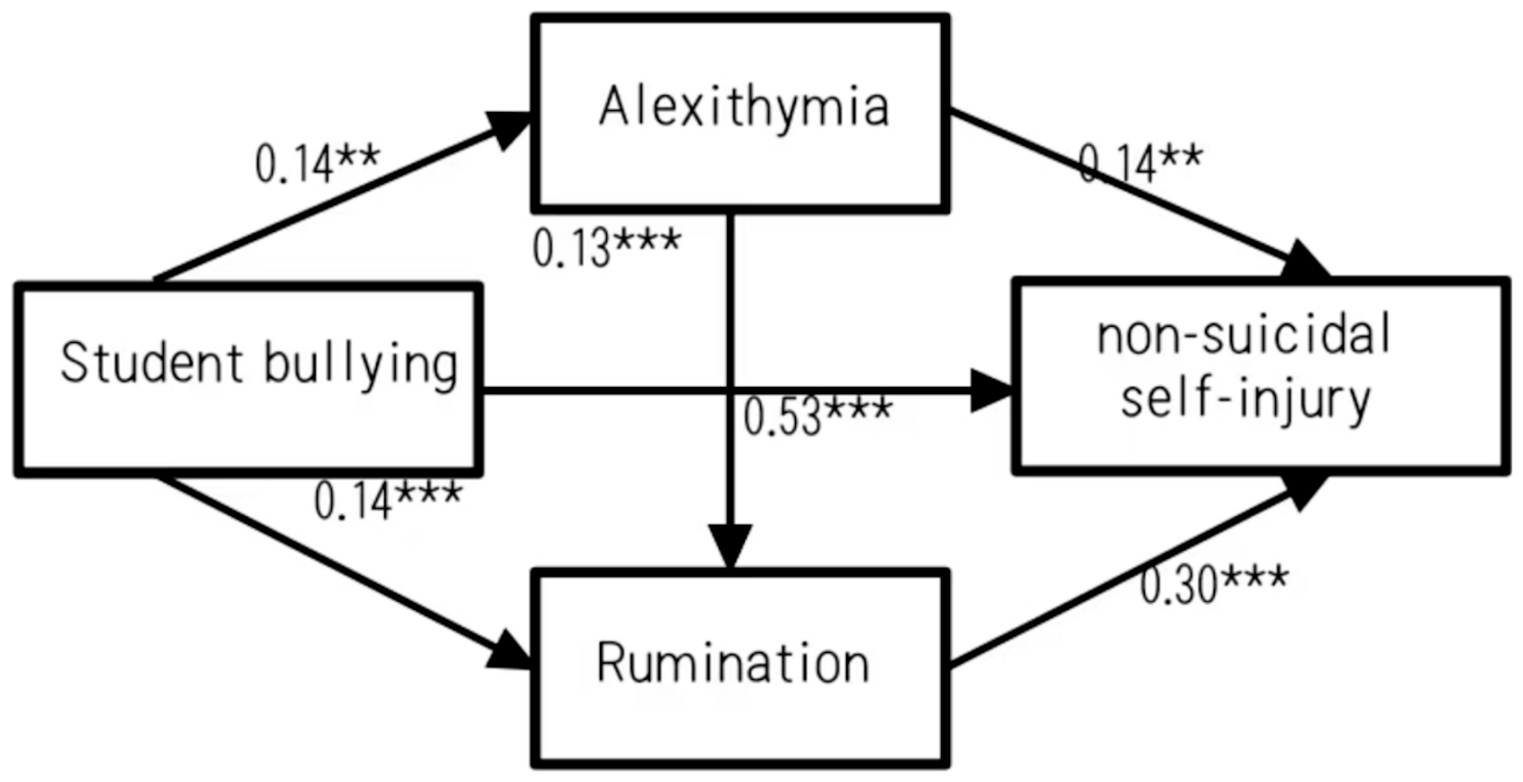
Chain mediation model actual path graph with data.

This study used structural equation modeling to test for the chain mediation effect to control measurement error. Variables were analyzed, and it was found that all predictor variables had variance inflation factors below 5, so there was no problem with multicollinearity. The total effect of student bullying on non-suicidal self-injury was significant (*β* = 0.21, SE = 0.46, t = 5.70, *p* = 0.000), controlling for gender and school year; secondly, two mediating variables - alexithymia and ruminative thinking - were added to the model to obtain the path model shown in Figure 2. It was found that the fitted model was good: *χ*/df = 3.11, RMSEA = 0.06, SRMR = 0.04, CFI = 0.97, TLI = 0.96.

**Figure 3:**
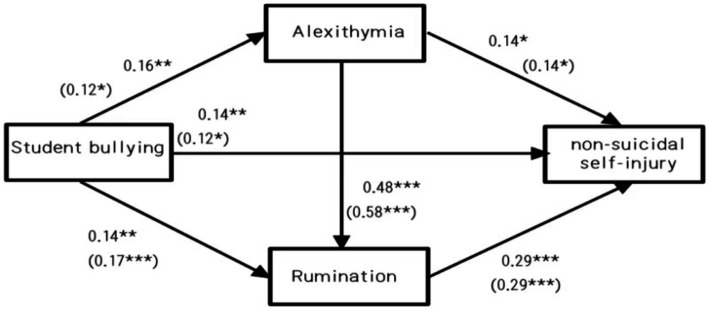
The actual path map with data in the chain-mediated model of different genders.

Student bullying can all successively affect non-suicidal self-injury through alexithymia and rumination, but there are some differences in their internal mechanisms.

In conclusion, Table 3 shows that the total effect of mediation is significant (b = 0.54, SE = 0.35, 95%CI = [0.14, 1.49]), and the direct effect of bullying on NSSI is significant (β = 0.34, SE = 0.27, 95%CI = [0.03,1.06]). The indirect effect of alexithymia (β = 0.05, SE = 0.04, 95%CI = [0.001,0.18]) and rumination (β = 0.08, SE = 0.05, 95%CI = [0.02,0.23]) was significant. And the indirect effects of alexithymia and rumination as chain mediators were significant (β = 0.07, SE = 0.06, 95%CI = [0.02,0.21]) Figure 3.

**Table 3:** Direct and indirect effects between latent variables.

Independent variable	Effect type	Path	SE	efficiency value	relative effect value	95% CI
Student bullying	Direct effect (β)	Student bullying→Non-suicidal self-injury	00.27	0.34	63.62%	[0.03,1.06]
	Indirect effect (β)	Student bullying→Alexithymia→Non-suicidal self-injury	00.04	0.05	80.40%	[0.001,0.18]
		Student bullying→Rumination→Non-suicidal self-injury	00.05	0.08	15.30%	[0.02,0.23]
		Student bullying→Alexithymia→Rumination→Non-suicidal self-injury	00.06	0.07	12.69%	[0.02,0.21]
	Total effect(β)		00.35	0.54	100%	[0.14, 1.49]

### 3.4 Tests for gender differences in mediation models

The present study examined whether there was cross-sex consistency in the mediating effects of alexithymia and rumination. First, the chained mediation effect models of bullying on non-suicidal self-injury among adolescent students were tested separately for boys and girls. ^22^The results showed that the fit indices for the boys’ model were: *χ*/df = 2.60, RMSEA = 0.07, SRMR = 0.06, CFI = 0.95, TLI = 0.94; and for the girls’ model were: χ/df = 1.92, RMSEA = 0.05, SRMR = 0.05, CFI = 0.97, TLI = 0.96. Overall, all the fit indices were consistent across genders. The fit indices were in the acceptable range for equal group comparisons. Subsequently, the method of multi-group comparison in structural equation modeling was used to set up the equivalent model, Model 1 (baseline model), defining that the boys’ and girls’ groups have the same model structure and the path coefficients are estimated freely; Model 2: restricting the corresponding path coefficients of the boys’ and girls’ groups to be equal based on Model 1. It was found that the fitting results of the two models were acceptable (χ/df = 2.18, RMSEA = 0.04, SRMR = 0.06, CFI = 0.96, TLI = 0.95; χ/df = 4.10, RMSEA = 0.07, SRMR = 0.07, CFI = 0.88, TLI = 0.88); moreover, the difference in fitting indices between the two models ΔCFI and ΔTLI were both greater than 0.01, which indicated significant gender differences (Jiang et al., 2023; Cheung and Rensvold, 2002; Kuo et al., 2024). With both male and female students significantly predicted by their respective chain mediators, the mediation effect value for female students (0.12) was twice as high as that for male students (0.06), suggesting that the chain mediators of rumination thinking and Alexithymia were more predictive for female students than for male students in the relationship between student bullying and non-suicidal self-injurious behaviors.

On this basis, the mediating roles and gender differences of alexithymia and rumination thinking between bullying and non-suicidal self-injury among students were examined. As shown in Table 4, both boys’ and girls’ chain mediation for both alexithymia and rumination thinking were significant as well (Boys:*β* = 0.06, SE = 0.07, 95%CI = [0.01,0.27]; Female: *β* = 0.12, SE = 0.21, 95%CI = [0.001,0.71]), and the paths mediated by alexithymia were non-significant; boys’ rumination thinking mediated the relationship between bullying and non-suicidal self-injury as compared to girls’ (*β* = 0.08, SE = 0.07, 95%CI = [0.01, 0.27]). Separate paths for girls’ alexithymia and rumination were found to be non-significant from the data, and only the overlap of both alexithymia and rumination played a role.

**Table 4:** Testing the mediating effect of male and female students.

Path	Male				Female			
	SE	efficiency value	relative effect value	95% CI	SE	efficiency value	relative effect value	95% CI
Student bullying→Alexithymia→Non-suicidal self-injury	0.07	0.04	8.49%	[−0.02,0.27]	0.21	0.05	7.75%	[−0.05,0.74]
Student bullying→Rumination→Non-suicidal self-injury	0.07	0.08	15.64%	[0.01,0.27]	0.34	0.15	21.76%	[−0.01,1.04]
Student bullying→Alexithymia→Rumination→Non-suicidal self-injury	0.07	0.06	11.58%	[0.01,0.27]	0.21	0.12	17.88%	[0.001,0.71]

## 4 Discussion

The present study found a two-by-two significant correlation between adolescent student bullying, alexithymia, rumination thinking, and nonsuicidal self-injury, suggesting that adolescents who students frequently bully are more likely to have alexithymia and rumination thinking and thus are more likely to engage in nonsuicidal self-injurious behaviors. Therefore, the internal mechanism of action between the variables was further examined based on the correlation analysis.

### 4.1 Bullying and non-suicidal self-injury

The results of the data indicated that adolescent student bullying significantly and positively predicted their non-suicidal self-injurious behaviors and Hypothesis H1 was confirmed, which is also consistent with previous studies. Several scholars have found a positive correlation between student bullying and non-suicidal behavioral self-injury (Baiden et al., 2017; Jiang et al., 2016; Claes et al., 2015). Some foreign scholars conducted a 1-year observational study on middle school students and found that being bullied by peers significantly predicted individuals’ non-suicidal self-injurious behavior (Jutengren et al., 2011). In addition, Moore et al. concluded that there is sufficient evidence to suggest a causal relationship between bullying and self-injury in childhood and adolescence (Moore et al., 2017). All of these studies provide a favorable basis.

In addition, the findings of this study also fit with the general stress theory and the interpersonal model. On the one hand, the general stress theory suggests that when an individual encounters environmental stimuli or stressful events (stressors), such as being bullied, and is unable to resolve or change their behavior to adapt to the demands of the environment, a subjective feeling of being oppressed is generated in their physiological psychology under such stressful stimuli. Thus, they are likely to alleviate or relieve the feeling of being oppressed through behaviors such as non-suicidal self-injury avoidance (Van Geel et al., 2015). On the other hand, Hilt suggests that non-suicidal self-injurious behaviors are ways of negatively coping with negative interpersonal relationships or events and that individuals experience changes in their interpersonal relationships with peers around them after experiencing a negative event of bullying (Hilt et al., 2008). The last tendency is to escape from difficult situations or to attract others for help through non-suicidal self-injurious behaviors (Muehlenkamp et al., 2013).

This outcome may be due to the rapid physical development of adolescents and their relative psychological immaturity, which makes it difficult for middle school students to adopt effective coping strategies when they are confronted with student bullying as a socially stressful event that cannot be resolved over a long period (Vacca et al., 2023) When the negative emotions are not released for an extended period, students will seek extreme ways to relieve their emotions or seek help, which will gradually strengthen the behavior and form a vicious circle in the long run, thus increasing the frequency of non-suicidal self-injurious behaviors. Therefore, teachers should pay more attention to the emotional and thinking problems of adolescents whom students have bullied, find out in time, and provide effective guidance to prevent bullying from causing more severe consequences.

### 4.2 The mediating role of alexithymia in bullying and non-suicidal relationships

Findings suggesting that student bullying can indirectly influence adolescents’ non-suicidal self-injurious behaviors through alexithymia are similar to those of Thomassin, who found that emotional expression mediates the relationship between emotional abuse and self-injurious behaviors in childhood (Thomassin et al., 2016). On the one hand, a small number of studies have investigated a positive correlation between alexithymia and bullying victimization (Önal Sönmez et al., 2020). Due to memory fragmentation and attentional lopsidedness in post-victimization individuals (Van der Kolk and Fisler, 1995), they have difficulty effectively utilizing their cognitive abilities. They have difficulty effectively utilizing their cognitive resources to process these emotional states and physiological arousal (Lischke et al., 2022). This may further exacerbate an individual’s difficulty in expressing emotions by causing bullying victims to have difficulty recognizing their own emotions and thus expressing and regulating traumatic emotions (Cowie and Berdondini, 2002). The more severe the student bullying, the higher the level of alexithymia. On the other hand, the traumatic experience serves as an external unpleasant stimulus because of prolonged exposure to the bullying event. Emotions may avoid unpleasant stimuli through non-suicidal self-injurious behaviors, creating negative reinforcement associations (Liu et al., 2022). Meanwhile, empirical studies have demonstrated that alexithymia directly predicts non-suicidal self-injurious behavior (Zhang et al., 2023).

The findings also fit with the social mechanism of alexithymia, which suggests that children’s exposure to certain undesirable social factors during their development, such as socially traumatic events like bullying, may cause severe damage to the individual’s psyche, which in turn reduces their level of socialization. As a result, the emotional regulation ability and self-evaluation system of those who experience bullying may be impaired and exhibit non-adaptive behaviors (Wang and Zhang, 2023).

### 4.3 The mediating role of rumination thinking in bullying and non-suicidal relationships

This study also found that ruminative thinking indirectly influences the relationship between student bullying and adolescent non-suicidal self-injurious behaviors, allowing hypothesis H3 to be confirmed, which is similar to the findings of Qin et al. (2024). Qian Qin concluded that rumination mediates the relationship between major life events and non-suicidal self-injury. On the one hand, the results are consistent with the expectations of the Stress Response Model, which posits that rumination is a subsequent response to a stressful event. When stressful events lead to negative outcomes, individuals tend to keep thinking about these negative outcomes (Robinson and Alloy, 2003). Bullying serves as an ongoing stressor (Kampoli et al., 2017). When individuals are unable to resolve and adapt, it can lead adolescents to become trapped in the negative effects of the bullying incident that are difficult to extricate themselves from, immersing themselves in distressing emotions, i.e., falling into rumination thinking. In the absence of effective adjustment strategies for the individual, compensatory behaviors such as non-suicidal self-injury may be adopted to restore personal and environmental balance in this way (Yusin, 1974; Agnew, 1992). Meanwhile, Malamut confirmed that bullying has a positive predictive effect on rumination thinking (Malamut and Salmivalli, 2021), supporting the present study’s findings.

On the other hand, the findings are consistent with the emotional cascade model, which suggests that ruminating on even small negative stimuli can exacerbate the experience of negative emotions (Selby et al., 2013). Rumination thinking manifests as a sustained focus on the negative stimulus and a repeated experience of the causes and consequences of the negative event. This persistent rumination further exacerbates negative emotions, and strong negative emotions may trigger more rumination, resulting in an emotional cascade (Selby et al., 2008). When rumination reaches a certain level where the individual is unable to cope with the psychological pain, the individual will use non-suicidal self-injury to alleviate their psychological pain (Qin et al., 2024). The following are some examples of non-suicidal self-injury. People report an increase in positive emotions in non-suicidal self-injury, which predicts more severe self-injury tendencies (Hasking et al., 2018), suggesting that non-suicidal self-injury is reinforced, making it a habitual way of coping. Meanwhile, several studies have confirmed that rumination directly predicts non-suicidal self-injury (Selby et al., 2010; Coleman et al., 2022), providing a basis for this study’s findings. Thus, rumination plays a partial mediating role in student bullying and non-suicidal self-injury.

### 4.4 Chain mediating role of alexithymia and ruminate thinking

The present study further found that alexithymia and rumination thought chain mediated the effects of student bullying on non-suicidal self-injury, and hypothesis H4 was supported. This finding illustrates that adolescents’ bullying experiences lead to individuals who are prone to difficulties in recognizing and expressing emotions and that individuals with high levels of alexithymia tend to have higher levels of rumination thinking and are ultimately more prone to non-suicidal self-injury. Specifically analyzed, student bullying leads individuals to develop high levels of alexithymia, and according to previous research, alexithymia, and rumination are two typical characteristics of depression (Taylor, 2000). The two are intrinsically congruent, and individuals with alexithymia tend to exhibit higher levels of rumination, which further diminishes their problem-solving and adaptive skills. To alleviate the resulting distress, individuals may engage in non-suicidal self-injurious behaviors.

On the one hand, goal progression theory suggests that when individuals are sensitive to unfulfilled goals, they tend to think about the emotional goals of the negative event repeatedly (Martin et al., 2003). On the one hand, Individuals with alexithymia have relatively little information to guide their behavior in emotional situations due to difficulties in identifying and expressing their own and others’ emotions. As a result, they may tend to think repetitively and analytically about the emotional goals triggered by bullying incidents (Wang and Zhang, 2023), i.e., to fall into rumination thinking, accumulate negative emotions, and ultimately release pain through non-suicidal self-injury. On the other hand, emotion recognition and expression can directly affect interpersonal situations (Kornreich, 2002). Difficulty in identifying and expressing emotions is a distinctive feature of people with alexithymia, which often leads to interpersonal tension, which in turn causes them to reduce their interactions with the outside world passively. In such situations, their thoughts and attention are more focused on internal emotions, and they repeatedly think about less unpleasant targets, i.e., ruminative thinking, which increases the risk of nonsuicidal self-injurious behavior. In addition, adolescents are in a sensitive period of physical and mental development, facing their changes, schooling, and various pressures in life, when they have insufficient ability to deal with problems and frequently experience negative emotions (Stephenson, 1985; Arnett, 1999). Therefore, when faced with bullying, they have difficulty adapting, accepting, or resolving the situation, as well as expressing their emotions. They can only think about the negative results repeatedly, which leads to negative emotions, and then non-suicidal self-injurious behaviors may occur. Moreover, Liu Yuxing and other scholars pointed out that alexithymia positively predicts rumination, and showed that alexithymia and rumination mediated the chain between childhood trauma and suicidal ideation in medical students (Liu et al., 2022). Which is similar to the hypothesis of the present study. Accordingly, affective alexithymia and rumination play a chain-mediating role in the relationship between bullying and self-injurious behaviors among students.

### 4.5 Gender differences in the mediation model

The data results indicate that there are significant gender differences in the chain mediation model regarding the impact of bullying on non-suicidal self-injury (NSSI), thus supporting Hypothesis H5. Specifically, the predictive power of the chain mediation model for females is significantly higher than that for males. Previous studies have shown that females exhibit significantly higher levels of rumination compared to males (Nolen-Hoeksema, 1987; Bugay, 2011). In particular, in response to negative events, females are more likely than males to cope by repeatedly thinking about and focusing on the potential causes of the events. Additionally, because females generally possess higher levels of empathy and emotional understanding (Espelage and Swearer, 2004), they may struggle to escape and effectively regulate these emotions.

However, existing research has indicated that the prevalence of alexithymia is higher among males or shows no significant gender difference (Carpenter and Addis, 2001; Viinikangas et al., 2009). This finding slightly contrasts with the results of the current study. There may be several reasons for this discrepancy:

First, the variations in the selection of variables between previous studies and this research may contribute to the differing findings, as this study included the variable of rumination. Given that different variables can influence gender effects in varying ways and directions, this may lead to conclusions that diverge from those of prior research.

Second, this study examines alexithymia and rumination only in a generalized manner, without delving deeper into the individual dimensions of alexithymia and rumination.

Lastly, this study is limited by its focus on a specific population, as it only surveyed rural adolescents, and the sample size was relatively small, which may weaken its representativeness. Additionally, the research method is confined to a cross-sectional design, lacking in-depth longitudinal analysis, which could explain the discrepancies between these results and those of earlier studies.

### 4.6 Significance of the present study

In summary, adolescents who experience student bullying are at risk for potential individual physical, mental, and social developmental problems. Bullied individuals may be more inclined to resort to non-suicidal self-injurious behaviors due to chronic psychological stress and unreleased negative emotions. This behavior affects an individual’s physical and mental health and may also negatively affect their social relationships, academic performance, and future development (Ttofi and Farrington, 2011). Student bullying is a significant impediment to an individual’s normal development, as previous research has found that adolescents who are frequently bullied are more likely than those who are not to experience a range of physical, psychological, and behavioral symptoms, including headaches, stomachaches, depression, non-suicidal self-injurious behaviors, and even suicidal behaviors (Rigby, 1998; John Calvin, 2023; Claes et al., 2015; Litwiller and Brausch, 2013). Therefore, it is necessary to deepen the research on the breadth and depth of student bullying to provide theoretical guidance for preventing and intervening in the phenomenon.

Through systematic analysis and evidence-based argumentation, this paper delves into the relationship between bullying experiences and non-suicidal self-injury (NSSI), proposing a new chained mediation model featuring alexithymia and rumination. This model enhances and expands empirical research in general strain theory (GST). According to GST, when individuals encounter stressful stimuli that they are unable to resolve or adapt to, they may experience forced subjective perceptions—such as difficulty recognizing, expressing, or regulating emotions, as well as persistent rumination on negative emotions triggered by stressful events. This intensifies the negative impact of bullying, ultimately leading individuals to maladaptive behaviors as a means to relieve the oppressive experience. By exploring the mediating roles of psychological factors such as alexithymia and rumination, this study enriches the conceptual understanding of the subjective experience of oppression, refines and extends general strain theory, and provides a new cognitive-emotional theoretical perspective and explanatory framework in this domain.

The practical significance of this study is particularly notable within the educational context. It not only aids schools and educators in gaining deeper insights into the roots, manifestations, and impacts of bullying and non-suicidal self-injury (NSSI) behaviors but also provides a scientific basis for developing and implementing effective school intervention or support programs. Through comprehensive research, schools can design more targeted prevention strategies, such as establishing anti-bullying mechanisms, conducting mental health education, and providing psychological counseling and support, thereby fostering a safe, respectful, and inclusive school environment.

### 4.7 Research implications

First, the study found that bullying successively affects non-suicidal self-injury through alexithymia and rumination, suggesting that socially traumatic experiences can lead to non-adaptive behaviors in bullied students, posing a threat to an individual’s physical and mental health. This effect is both severe and far-reaching, so parents and schools should pay more attention to the physical and mental health of the bullied. Secondly, more mental health education courses should be introduced in schools to popularize anti-bullying knowledge and make students aware of the seriousness of bullying. At the same time, students should be guided to look at themselves correctly, cope with negative events positively, and promote the positive development of emotion regulation and cognitive attribution to enhance adaptive ability. In addition, as different genders and individuals show different ways of coping with bullying among students, we should pay attention to each individual without discrimination and provide personalized counseling according to different situations.

Furthermore, given that bullying is a negative interpersonal event, teachers should always pay attention to interpersonal interactions within the classroom and properly manage interpersonal relationships among students. Instant education should be provided to the bullies, immediate guidance should be given to the bullied, and correct guidance should be given to the bystanders. Adolescent students face multiple pressures and rapid physical and mental development. Schools can organize outdoor activities to help students relax and promote the harmonious development of interpersonal relationships. Finally, previous studies have mostly focused on common emotional problems such as depression, anxiety, and insomnia in bullied students but less on aspects such as alexithymia and rumination. Therefore, by delving into other aspects of cognitive emotions, we can gain a more comprehensive understanding of the process of bullying’s impact on adolescents and provide solid theoretical support for bullying intervention.

### 4.8 Limitations and prospects

Although this study has yielded a series of valuable findings and has made contributions to advancing knowledge in related fields, it also recognizes several significant limitations that need to be addressed in future research endeavors.

Firstly, the study focused exclusively on rural middle school students as the research subjects, which somewhat restricts the generalizability and applicability of the findings. To provide a more comprehensive understanding of the issue, future research could consider including urban middle school students as a comparative control group. This would enrich the content and results of the study, making the conclusions more representative and persuasive.

Secondly, the study employed a cross-sectional design, which, while effective in revealing associations between variables at a single point in time, limits the exploration of the dynamic relationships among variables over time. To gain deeper insights into the intrinsic connections between variables and to confirm the reliability of the findings, future studies should adopt longitudinal tracking methods. This would involve prolonged observation and recording to uncover the changes and interrelations among variables across time.

Thirdly, the evaluation in this study was conducted by teachers, which introduces the possibility of bias, such as social desirability effects. Despite ensuring anonymity and providing training for the teachers to follow standardized procedures, the presence of teachers during the assessment may have influenced students’ responses. This limitation should be considered when interpreting the findings. Future research should aim to minimize this type of bias by employing external assessors or online survey methods to enhance the accuracy and objectivity of the data.

Fourthly, this study did not include an evaluation of clinical diagnoses such as eating disorders, borderline personality disorder, or other psychiatric conditions that are known to significantly increase the risk of non-suicidal self-injury (NSSI). While efforts were made to control for certain psychological variables, the absence of detailed diagnostic data limits the ability to comprehensively interpret the findings. Future research should incorporate structured diagnostic interviews or validated clinical screening tools to better account for the influence of these psychiatric factors and provide a more nuanced understanding of the predictors of NSSI.

Lastly, while this research primarily focused on exploring chain mediation effects, which provides new insights into the relationship between bullying and self-injurious behaviors, the content of the study still requires further expansion. Future investigations could consider including moderation effects to examine how other factors influence the chain mediation process, providing more comprehensive and in-depth theoretical support for interventions targeting bullying. This would in turn help to more effectively prevent and reduce occurrences of adolescent self-injurious behaviors.

Furthermore, future research should not only deepen theoretical exploration but also actively incorporate practical intervention studies as an essential component to further enrich and enhance the research content. By designing and implementing a range of scientifically sound practical intervention measures, it could create a more positive, healthy, and uplifting learning atmosphere for adolescents, effectively promoting their physical and mental well-being. Such practical intervention research would not only validate the effectiveness and feasibility of theoretical hypotheses but also provide valuable practical experience and reference for educators, parents, and policymakers, thus collectively contributing to the healthy growth of adolescents.

## 5 Conclusion

1. Bullying experiences significantly predict non-suicidal self-injurious behaviors among rural adolescents.
2. Alexithymia and rumination play a chain mediating role in the relationship between bullying and non-suicidal self-injury, forming three paths: (i) Alexithymia mediates the relationship between bullying and non-suicidal self-injury among rural adolescents; (ii) Rumination mediates the relationship between bullying and non-suicidal self-injury among rural adolescents; (iii) There is a chain effect of alexithymia and rumination in the relationship between bullying and non-suicidal self-injury.
3. There are significant gender differences in the chain mediation model between bullying and non-suicidal self-injury, with the predictive power of rumination and alexithymia as mediators being significantly greater for females than for males.

## Data Availability

The datasets presented in this study can be found in online repositories. The names of the repository/repositories and accession number(s) can be found below: https://pan.baidu.com/s/1XoskIOZ-w0ZV8UpeBfiaZA?pwd=vvr9, Code: vvr9.
